# Artificial Intelligence for Assessment and Feedback in Medical Education: Bibliometric Mapping Study and Thematic Evidence Map

**DOI:** 10.2196/98949

**Published:** 2026-07-02

**Authors:** Zihang Zhao, Zihan Liu, Liang Guo, Teng Pan, Yousheng Zhang, Chenxiang Miao, Yiting Ge, Yipeng Wang, Xin Hu, Xin Wang, Ruipeng Zhang, Zhiyong Hou

**Affiliations:** 1 Department of Orthopedic Surgery The Third Hospital of Hebei Medical University Shijiazhuang, Hebei China; 2 Hebei Medical University Shijiazhuang, Hebei China; 3 Department of Oncology Shijiazhuang People’s Hospital Shijiazhuang, Hebei China; 4 Faculty of Medicine, Dentistry and Health Sciences The University of Melbourne Melbourne, Victoria Australia

**Keywords:** artificial intelligence, generative artificial intelligence, large language model, medical education, learner assessment, feedback, bibliometric analysis, evidence map, summative assessment, validity, fairness, governance

## Abstract

**Background:**

Artificial intelligence (AI), particularly generative AI and large language models, is increasingly used for assessment-related tasks in medical education. Existing overviews often address AI in medical education broadly, limiting assessment-specific interpretation of functions, settings, learner stages, and responsible-AI reporting domains.

**Objective:**

This study aims to map the literature on AI for assessment and feedback in medical education, including publication trends, bibliometric structure, assessment functions, AI types, settings, learner stages, and reporting of validity, reliability, fairness, integrity, transparency, human oversight, implementation, and governance.

**Methods:**

We conducted a bibliometric mapping study incorporating structured thematic evidence-map coding. Web of Science Core Collection, Scopus, and PubMed were searched from January 1, 2015, to April 8, 2026. Document selection and main evidence-map coding were based primarily on titles, abstracts, and bibliographic metadata, with targeted ambiguity resolution. Because reporting domains may appear mainly in full-text sections, all 435 included records underwent full-text sensitivity analysis for the 8 reporting domains. Coding reliability was assessed by two coders using percent agreement and Cohen κ before adjudication. Exploratory subgroup analyses and an excluding-2026 partial-year sensitivity analysis were conducted.

**Results:**

Searches identified 14,968 records; 435 were included after deduplication and selection. Overall, 399 (91.7%) records were indexed in the post-ChatGPT period. Generative AI was coded in 310 (71.3%) records, and large language models in 301 (69.2%) records. In the assessment-function umbrella analysis, learner performance evaluation accounted for 270 (62.1%) records, feedback for 93 (21.4%) records, assessment content generation for 65 (14.9%) records, and other or unclear functions for 7 (1.6%) records. The most common settings were board-style examinations (n=151, 34.7%) and written examinations (n=88, 20.2%); undergraduate medical education was the most represented learner stage (n=172, 39.5%). Full-text–confirmed reporting was most frequent for reliability (n=288, 66.2%) and implementation (n=231, 53.1%); intermediate for validity (n=158, 36.3%), fairness (n=132, 30.3%), and transparency (n=130, 29.9%); and less frequent for governance (n=57, 13.1%), human oversight (n=46, 10.6%), and integrity (n=26, 6%). Stage 1 κ values ranged from 0.785 to 0.895, and stage 2 κ values ranged from 0.809 to 0.880. Excluding 65 partial-year 2026 records did not change the overall interpretation.

**Conclusions:**

The indexed English-language literature on AI for assessment and feedback in medical education expanded rapidly in the post-ChatGPT period and was concentrated in generative AI, large language models, examination-oriented assessment, and undergraduate medical education. Future studies should complement examination benchmarking with authentic assessment contexts, distinguish assessment content generation from learner-facing evaluation and feedback, and report responsible assessment domains more consistently.

## Introduction

Artificial intelligence (AI) is increasingly shaping medical education through learning analytics, simulation, adaptive learning, clinical reasoning support, assessment, feedback, and faculty development [1-4]. Earlier applications often involved machine learning, deep learning, natural language processing, computer vision, or rule-based systems applied to defined educational or clinical-learning tasks [1,2,4,5]. More recently, generative artificial intelligence (GenAI) and large language models have changed the field because they can generate explanations, assessment items, simulated dialogue, feedback, and other educational content with relatively low technical barriers for educators and learners [6-10].

Assessment and feedback deserve focused attention within this broader literature because assessment in medical education is not only a measurement activity [11]. It supports learning, informs feedback, contributes to judgments of competence, and may influence progression, remediation, certification, and professional trust [12-15]. When AI is used to generate assessment materials, score responses, interpret performance, or provide feedback, the relevant concerns extend beyond technical accuracy to include validity, reliability, fairness, academic integrity, transparency, human oversight, implementation, accountability, and governance [16-20].

Existing reviews and bibliometric studies have provided valuable overviews of AI in medical education and health professions education [3,6,21,22]. These broad syntheses may combine studies of curriculum, learning support, simulation, attitudes toward AI, assessment, institutional readiness, and faculty development. As a result, they may not isolate which assessment functions are being studied, which learner stages and settings are represented, and how often assessment-quality or governance domains are explicitly reported. This distinction has become more important in the GenAI era, when examination benchmarking, question generation, automated feedback, and performance interpretation may appear within the same broad literature but carry different educational implications.

A further distinction is needed between assessment content generation and learner-facing evaluation or feedback. A study that uses AI to generate multiple-choice questions, a study that evaluates a model’s performance on licensing-style examinations, and a study that uses AI to support feedback on learner performance all relate to assessment, but they do not answer the same educational question. Content generation concerns the production or refinement of assessment materials, whereas learner-facing assessment involves scoring, performance evaluation, interpretation, feedback, or decisions about learner progress. This distinction follows from assessment theory, which links evidence requirements to intended interpretations, uses, and consequences [11-13,15].

This study aimed to map the literature on AI for assessment and feedback in medical education through a bibliometric mapping study and thematic evidence map. Specifically, we examined temporal publication trends, bibliometric structure, assessment functions, AI types, assessment settings, learner stages, and reporting of validity, reliability, fairness, integrity, transparency, human oversight, implementation, and governance. We also conducted a full-text sensitivity analysis for these reporting domains, assessed 2-stage coding reliability, disaggregated assessment content generation from learner-facing assessment functions, and evaluated exploratory subgroup and partial-year sensitivity patterns.

## Methods

### Study Design and Reporting Framework

We conducted a bibliometric mapping study incorporating structured thematic evidence-map coding to characterize the literature on AI for assessment and feedback in medical education [23-25]. The design emphasizes field characterization, including publication trends, bibliometric structure, assessment functions, settings, learner stages, AI categories, and reporting domains. Reporting was guided by the BIBLIO (bibliometric reviews of biomedical literature) checklist, where applicable [26], with additional evidence-map and review-methodology references used to support the structured thematic evidence-map component [24,25]. The completed BIBLIO checklist is provided in Multimedia Appendix 1. Because this study was designed as a bibliometric mapping study, no protocol was registered.

The analytic unit was the screened bibliographic record. Document selection and the main evidence-map coding were conducted primarily from titles, abstracts, and bibliographic metadata, with targeted ambiguity resolution when needed. Because reporting domains such as fairness, integrity, transparency, human oversight, implementation, and governance may appear primarily in full-text sections, we conducted a full-text sensitivity analysis for the 8 reporting domains across all 435 included records.

Methodological transparency was supported through a 2-layer reporting structure in which the main manuscript describes the study design and analytic workflow, and the supplementary materials provide database-specific search strategies, coding definitions, sensitivity analyses, agreement tables, and bibliometric-parameter settings.

### Data Sources and Search Strategy

We searched Web of Science Core Collection, Scopus, and PubMed from January 1, 2015, to April 8, 2026. The 3-database strategy was selected to balance multidisciplinary citation indexing, broad scholarly coverage, and biomedical indexing relevant to medical education, assessment, and AI.

The search strategy combined terms related to AI, including machine learning, deep learning, GenAI, large language models, ChatGPT, GPT, foundation models, and language models; assessment and feedback, including scoring, grading, examinations, testing, objective structured clinical examinations, item generation, question generation, performance evaluation, automated scoring, formative assessment, and summative assessment; and medical education, including medical students, undergraduate medical education, graduate medical education, residency, clerkship, internship, continuing medical education, and continuing professional development. Database-specific search strings, limits, and final hit counts are provided in Multimedia Appendix 2.

The final searches identified 14,968 records: 4381 from Web of Science Core Collection, 7256 from Scopus, and 3331 from PubMed.

### Eligibility Criteria

Records were eligible if they addressed AI-supported assessment or feedback in a medical education context. Assessment was defined as any AI-supported activity used to generate, score, interpret, or provide feedback on learner performance or assessment content in a medical education context.

Eligible records addressed learner assessment, performance evaluation, automated scoring, feedback, examination activity, item generation, question generation, formative or summative assessment, interpretation of learner performance, or related assessment processes. Eligible educational populations included medical students, residents, graduate medical trainees, physicians, clinicians, continuing medical education participants, or mixed medical education learner groups. Eligible publication types included original research, validation studies, reviews, scoping reviews, systematic reviews, bibliometric or mapping studies, and mixed methods studies.

We excluded records focused only on clinical diagnosis, prognosis, image interpretation, clinical prediction, or decision support without a medical education assessment or feedback context. We also excluded records focused only on teaching support, curriculum delivery, admissions, recruitment, patient education, nonmedical learner populations, or general AI commentary without an assessment or feedback component. Nonanalytic publication types, including conference abstracts, editorials, news items, and letters, were excluded. The search was limited to English-language records indexed in the selected databases.

### Deduplication and Document Selection

Database exports were merged and deduplicated through multistage matching based on DOI, PubMed identifier, normalized title, publication year, and available bibliographic metadata. After multistage deduplication, 8378 records remained for document selection.

Document selection was conducted primarily at the title and abstract level because the analytic unit was the screened bibliographic record, and the study aimed to map the structure and characteristics of a large assessment-focused literature. Bibliographic metadata were used to support record identification and classification. For records with insufficient title or abstract information, targeted ambiguity resolution was conducted through supplementary web-assisted review and, when necessary, PDF-assisted review. This ambiguity-resolution process was used to clarify eligibility and main evidence-map categories while preserving the bibliographic-record-based mapping design.

Following document selection, 7943 records were excluded, and 435 records comprised the final screened analysis cohort.

### Data Extraction and Evidence-Map Coding

For each included record, we extracted bibliometric metadata including publication year, authors, journal, DOI, country, and institution information derived from author affiliations, keywords, cited references, and citation-related fields used for bibliometric visualization.

We then applied a structured evidence-map coding framework to classify publication type, study design, AI type, assessment function, assessment-function umbrella, assessment setting, learner stage, GenAI relevance, post-ChatGPT period, and reporting domains visible in titles or abstracts. AI type categories included large language model, GenAI, machine learning, deep learning, natural language processing, computer vision, rule-based or expert systems, wearable or sensor-based AI, and other AI approaches.

Assessment function was coded using nonmutually exclusive categories, including formative assessment, summative assessment, feedback, automated scoring, performance evaluation, interpretation of learner performance, item generation, question generation, and other functions. To address conceptual overlap, we also coded an assessment-function umbrella variable distinguishing learner performance evaluation, feedback, assessment content generation, and other or unclear functions. Question generation and item generation were treated as assessment content generation and were not automatically coded as summative assessment unless the record explicitly linked them to examination use, learner performance evaluation, or assessment decision-making.

Assessment setting and learner stage were coded separately. Learner-stage categories included undergraduate medical education, residency, graduate medical education, physician or clinician continuing education, continuing medical education, mixed or multiple learner stages, and unclear. Because individual records could involve more than 1 AI type, assessment function, or assessment setting, evidence-map categories were treated as nonmutually exclusive unless otherwise specified. Additional coding definitions and operational details are provided in Multimedia Appendix 3.

### Full-Text Sensitivity Analysis for Reporting Domains

The main evidence-map categories remained based on the final manually reviewed coding based on titles and abstracts, with targeted ambiguity resolution. The initial reporting-domain variables captured explicit reporting visible in titles or abstracts of validity, reliability, fairness, integrity, transparency, human oversight, implementation, and governance. These estimates, based on titles and abstracts, were retained as the baseline.

Because these domains may be discussed mainly in full-text Methods, Results, Discussion, Limitations, tables, figures, supplementary materials, or governance-related sections, we conducted a full-text sensitivity analysis for all 8 reporting domains across all 435 included records. A domain was coded as present only when the full text explicitly reported, discussed, evaluated, or proposed considerations relevant to that domain. Coding was conservative; domains were not inferred from the topic, method, or study design alone. If the correct full text could not be verified or was unavailable, domain status was coded as unclear and not inferred.

Integrity was defined as explicit attention to academic integrity, assessment security, or safeguards against misuse of AI in assessment contexts. Human oversight was defined as explicit attention to human review, supervision, escalation, or educator involvement in AI-supported assessment or feedback processes. Transparency was defined as explicit reporting or discussion of model explainability, disclosure, interpretability, model reporting, or transparency of AI-supported assessment processes. Governance was defined as explicit attention to policy, accountability, regulation, institutional governance, privacy or data governance, or formal guidance for responsible AI use.

Full-text–confirmed estimates were used as the primary basis for interpreting reporting-domain findings. Estimates based on titles and abstracts were reported as the baseline and sensitivity comparison.

### Coding Reliability and Adjudication

We assessed coding reliability through a 2-stage independent coding process across the full cohort of 435 records. Two coders used the same codebook. Before independent coding, the coders reviewed the codebook and coding examples to calibrate the interpretation of category definitions. In stage 1, the coders independently coded the evidence-map categories coded from titles and abstracts using available bibliographic information. Stage 1 variables included publication type, study design, AI type exact set, assessment function exact set, assessment-function umbrella, assessment setting, learner stage, GenAI relevance, and post-ChatGPT period.

In Stage 2, the 2 coders independently coded the 8 full-text reporting domains: validity, reliability, fairness, integrity, transparency, human oversight, implementation, and governance. Agreement was calculated before adjudication using percent agreement and Cohen κ. For multilabel variables, exact-set agreement was used. Disagreements were resolved through consensus review against the codebook and source record, and final adjudicated codes were assigned after discussion. For low-prevalence variables, including integrity and human oversight, Cohen κ was interpreted alongside percent agreement because prevalence can affect κ estimates. Detailed agreement tables are provided in Multimedia Appendix 3.

### Bibliometric and Evidence-Map Analysis

We summarized annual publication counts, publication types, study designs, AI types, assessment functions, assessment settings, learner stages, GenAI relevance, and post-ChatGPT status using counts and percentages. The post-ChatGPT period was defined operationally as records indexed from November 2022 onward, corresponding to the public release of ChatGPT.

Bibliometric analyses characterized journal output, citation patterns, country-level collaboration, institutional contribution, author contribution, keyword structure, thematic evolution, and recent co-citation structure. Country and institution fields were derived from author affiliation metadata after harmonization. Country-level findings were interpreted as patterns within the indexed English-language records captured by the search, not complete global research output. Weighted author metrics were used to reduce inflation from large multiauthor records. Bibliometric software, thresholds, normalization, clustering, layout, counting, and weighting parameters are reported in Multimedia Appendix 3.

Evidence maps were constructed for the assessment function by AI type and assessment setting by learner stage. Because evidence-map categories were nonmutually exclusive, matrix counts represent coded study characteristics, not mutually exclusive study strata.

### Subgroup and Partial-Year Sensitivity Analyses

We conducted exploratory subgroup analyses for full-text–confirmed reporting domains by GenAI relevance, post-ChatGPT period, and assessment content generation status. Fisher exact tests were used for subgroup comparisons, with Benjamini-Hochberg correction for multiplicity. All 8 reporting domains were retained in the supplementary subgroup tables. In the main text, subgroup findings are reported selectively and interpreted cautiously as descriptive patterns.

Because 2026 was incomplete at the time of the search, annual trend figures identify 2026 as a partial year through April 8, 2026. We also conducted an excluding-2026 sensitivity analysis to evaluate whether the main reporting-domain patterns were robust to the removal of partial-year records. Detailed subgroup and partial-year sensitivity results are provided in Multimedia Appendix 3.

### Ethical Considerations

This study analyzed bibliographic records and published reports and did not involve human participants, patients, private identifiable information, or intervention delivery. Ethics review and informed consent were not required.

## Results

### Study Selection and Temporal Growth

**Figure 1:**
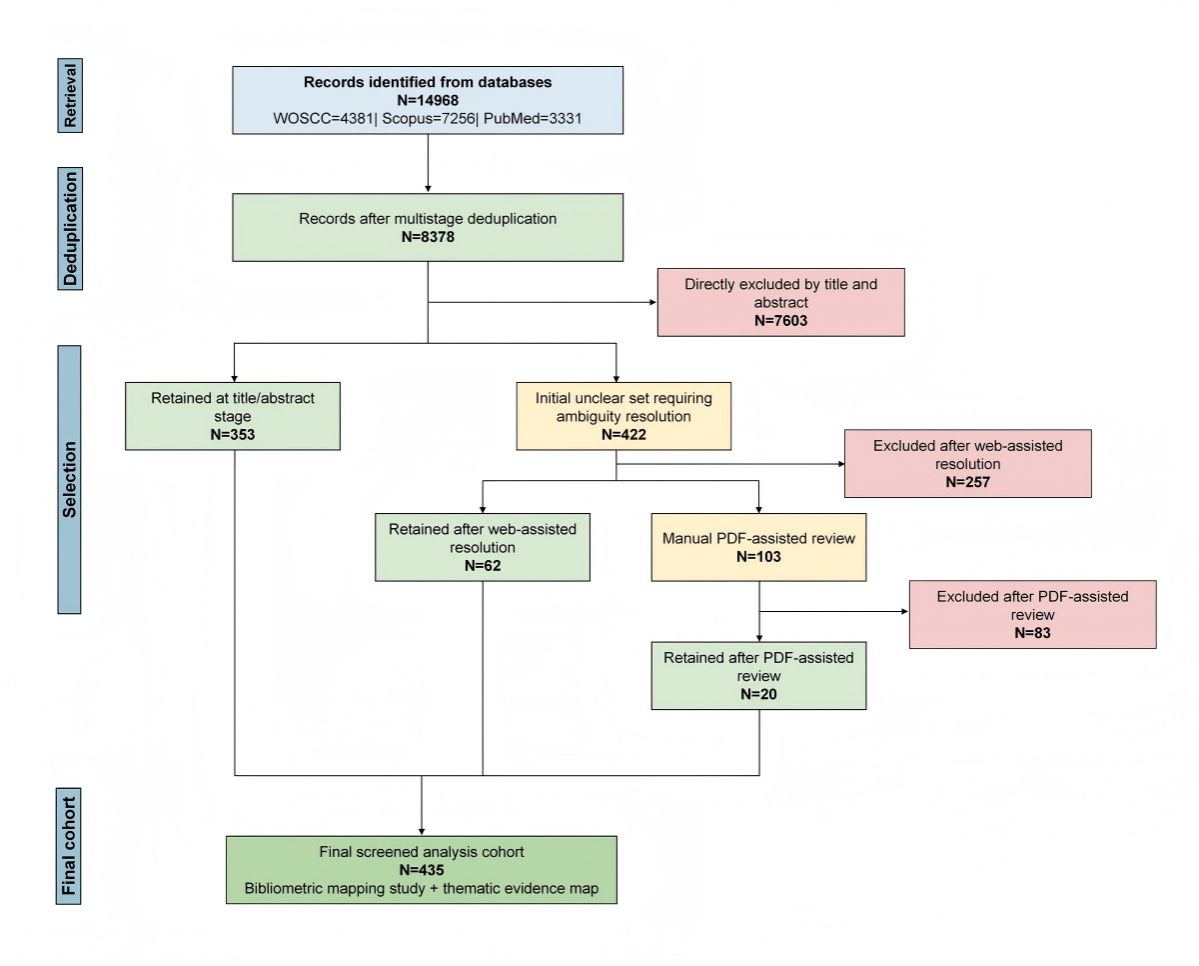
Study selection flow diagram. Records were identified from Web of Science Core Collection, Scopus, and PubMed; deduplicated through a multistage matching workflow; and screened during document selection. The final cohort included records retained at the title and abstract stage, after web-assisted ambiguity resolution, and after manual PDF-assisted review. The diagram accounts for all 8378 records entering document selection, comprising 7943 exclusions and 435 included records.

The database searches identified 14,968 records, including 4381 from Web of Science Core Collection, 7256 from Scopus, and 3331 from PubMed. After multistage deduplication, 8378 records remained for document selection. During document selection, 7943 records were excluded, leaving 435 records in the final screened analysis cohort (Figure 1).

**Figure 2:**
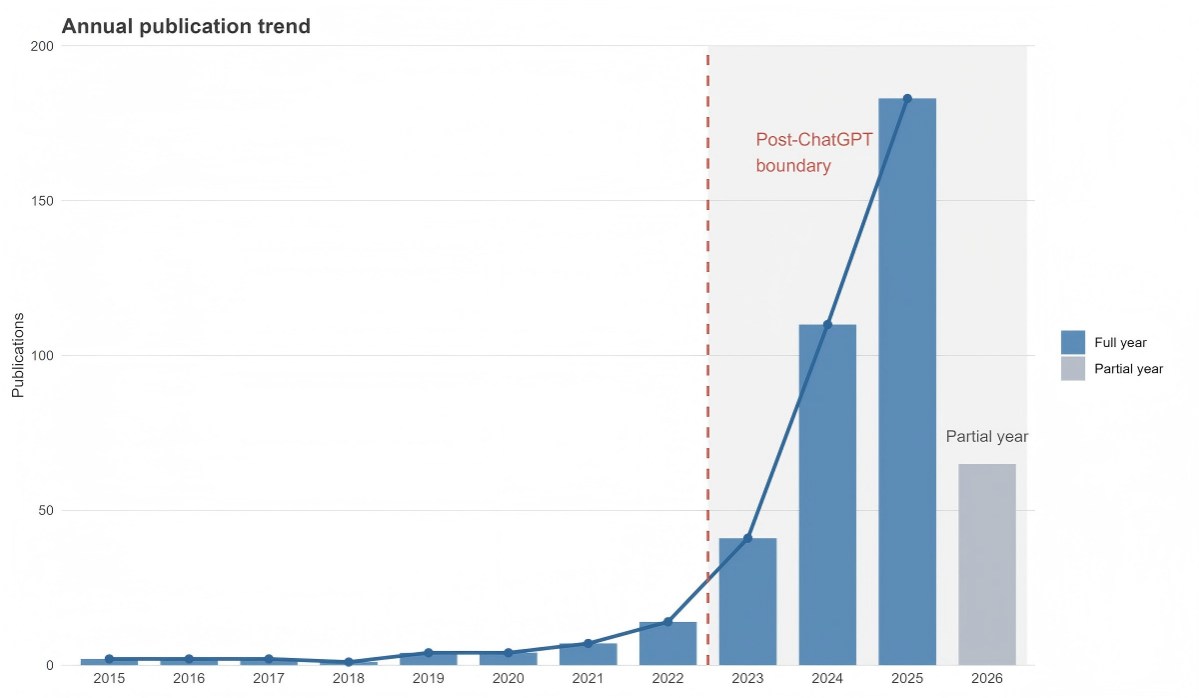
Annual publication trend in artificial intelligence for assessment and feedback in medical education, 2015-2026. Bars show the number of records by publication year. The shaded region denotes the post-ChatGPT period, operationally defined as records indexed from November 2022 onward. The line connects complete calendar years through 2025. The year 2026 is shown as a partial year through the final search date of April 8, 2026.

Original research accounted for 334 (76.8%) records, followed by validation studies (n=46, 10.6%), reviews (n=25, 5.7%), and mixed methods studies (n=19, 4.4%). Annual output remained low through 2021, with 2 records in 2015, 2 in 2016, 2 in 2017, 1 in 2018, 4 in 2019, 4 in 2020, and 7 in 2021. Output then increased to 14 records in 2022, 41 in 2023, 110 in 2024, and 183 in 2025. By the search date of April 8, 2026, 65 records had been indexed in 2026; this year was treated as a partial year. Overall, 399 (91.7%) records were indexed in the post-ChatGPT period, defined as November 2022 onward (Figure 2).

### Bibliometric Structure and Publication Characteristics

**Figure 3:**
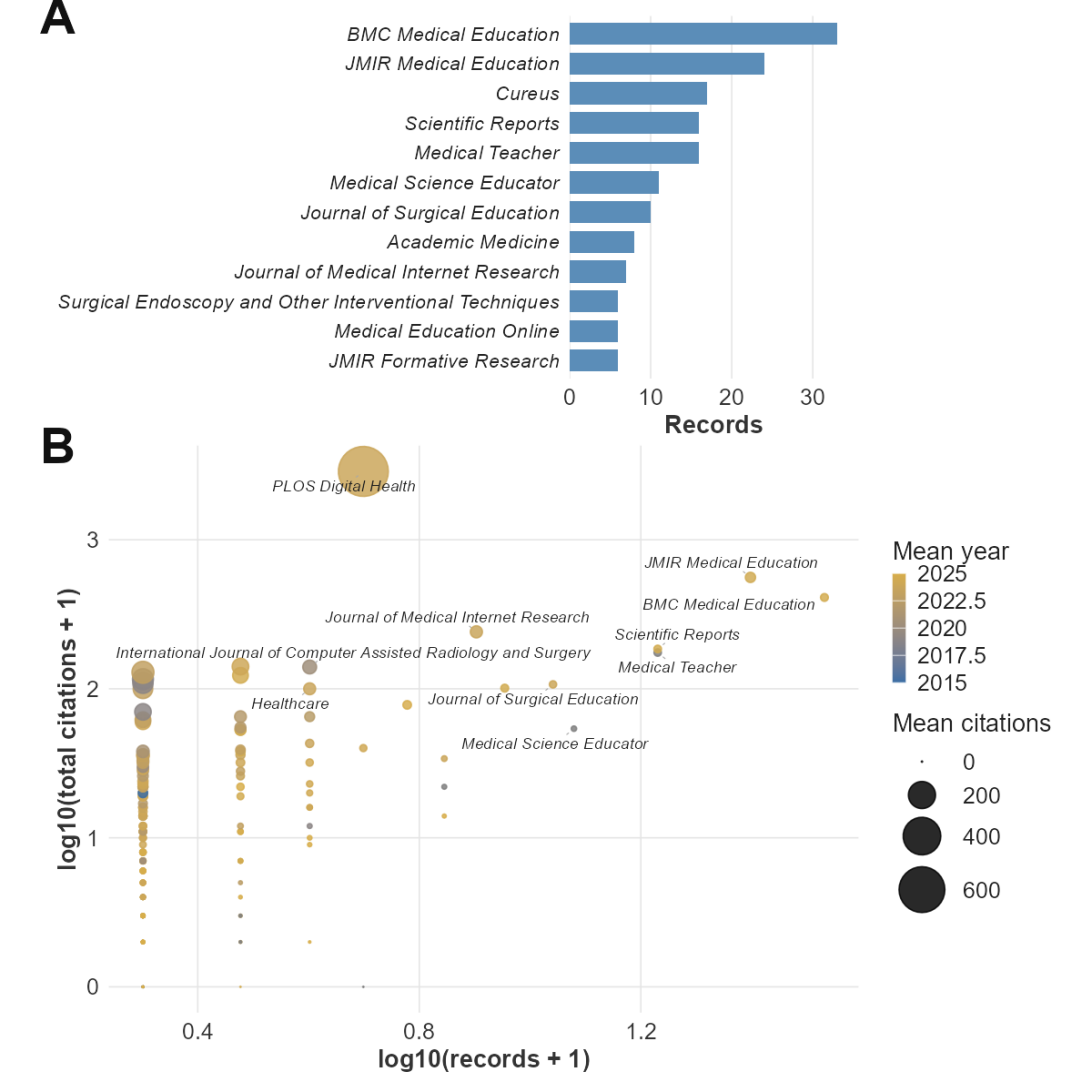
Journal landscape of the final screened analysis cohort. Panel A shows the leading journals by publication count. Panel B plots journal output against citation indicators, with bubble size representing average citations per record and color indicating mean publication year. Counts and citation indicators reflect indexed English-language records captured by the search and should be interpreted descriptively.

The final screened analysis cohort was distributed across a broad journal base, with output concentrated in medical education, digital health, and specialty education journals (Figure 3). Based on the final manually reviewed coding worksheet, the leading journals by record count were *BMC Medical Education* (n=32, 7.4%), *JMIR Medical Education* (n=24, 5.5%), *Cureus* (n=17, 3.9%), *Scientific Reports* (n=16, 3.7%), and *Medical Teacher* (n=16, 3.7%). In the journal landscape, *JMIR Medical Education* and *BMC Medical Education* combined visible publication volume with citation activity. *Journal of Medical Internet Research* and *PLOS Digital Health* had smaller record counts but high citation indicators, reflecting the visibility of highly cited digital health and large language model assessment papers. These citation indicators should be interpreted cautiously because publication timing and early benchmark visibility can strongly influence citation accumulation.

**Figure 4:**
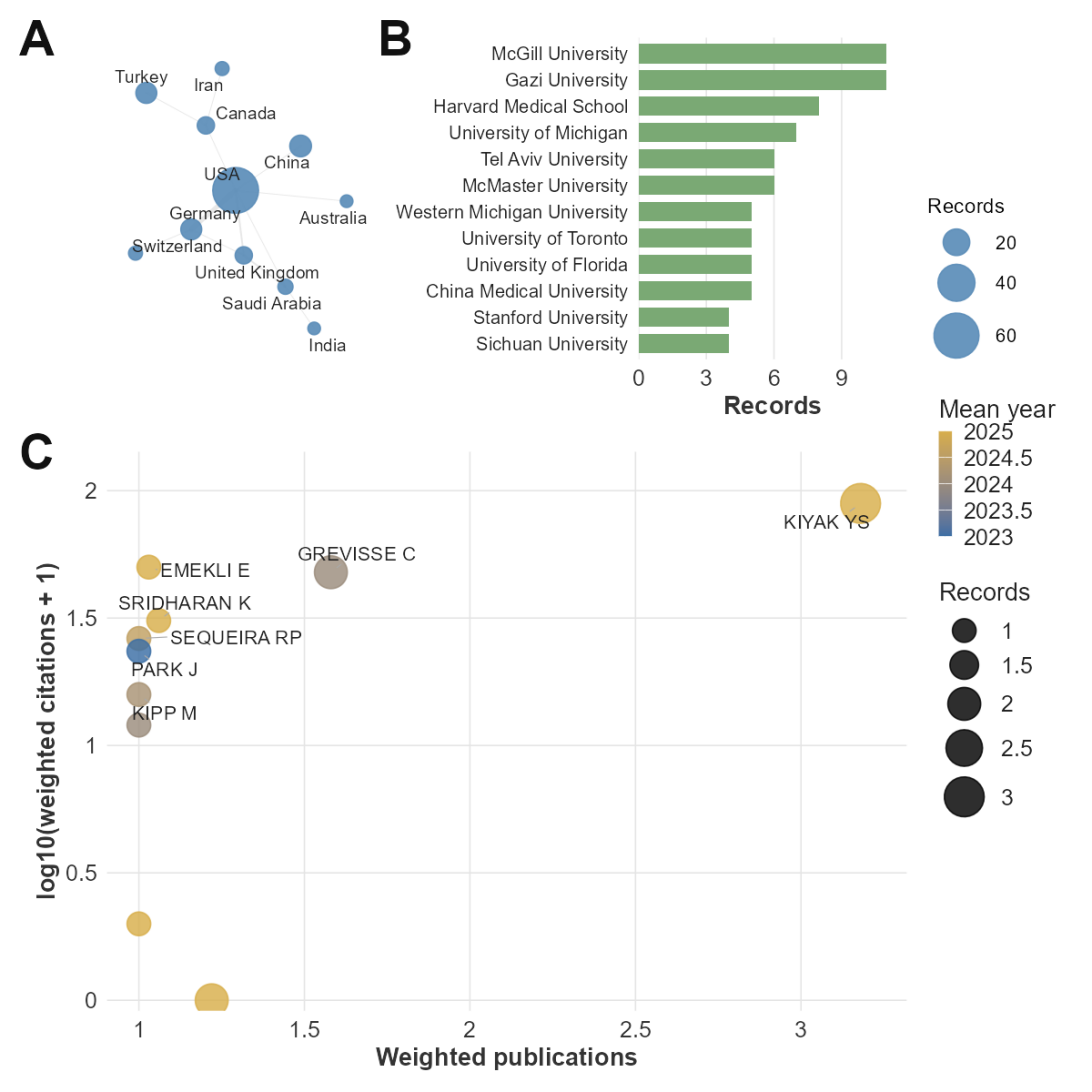
Collaboration and contributor structure in the indexed English-language corpus. Panel A shows the country-level collaboration network, with node size proportional to record count and line thickness proportional to collaboration strength. Panel B shows leading institutions by record count. Panel C shows leading authors using weighted publication output and citation profile, with color indicating mean publication year. Country, institution, and author patterns reflect records captured by the search and do not represent complete global output.

Country-level collaboration was interpreted as a pattern within the indexed English-language records captured by the search, not as complete global research output (Figure 4). Within this indexed corpus, the United States was the largest contributing country and the main collaboration hub in the coauthorship network, with 62 (14.3%) records. China (n=13, 3%), Germany (n=12, 2.8%), and Turkey (n=12, 2.8%) were the next most represented countries. The collaboration network indicated international linkage among several leading contributors, but the English-language and database-indexing restrictions limit conclusions about global research capacity or regional participation.

Institution and author summaries were retained as descriptive bibliometric indicators. In the supplementary institution table, McGill University, Gazi University, and the University of Michigan appeared among the leading institution entries. In the weighted author landscape, Kiyak YS showed the strongest weighted publication profile, with Grevisse C and Emekli E also appearing prominently in the combined output and citation space. These author- and institution-level findings should be interpreted cautiously because publication timing, team size, affiliation-string harmonization, and citation accumulation can influence bibliometric visibility.

**Figure 5:**
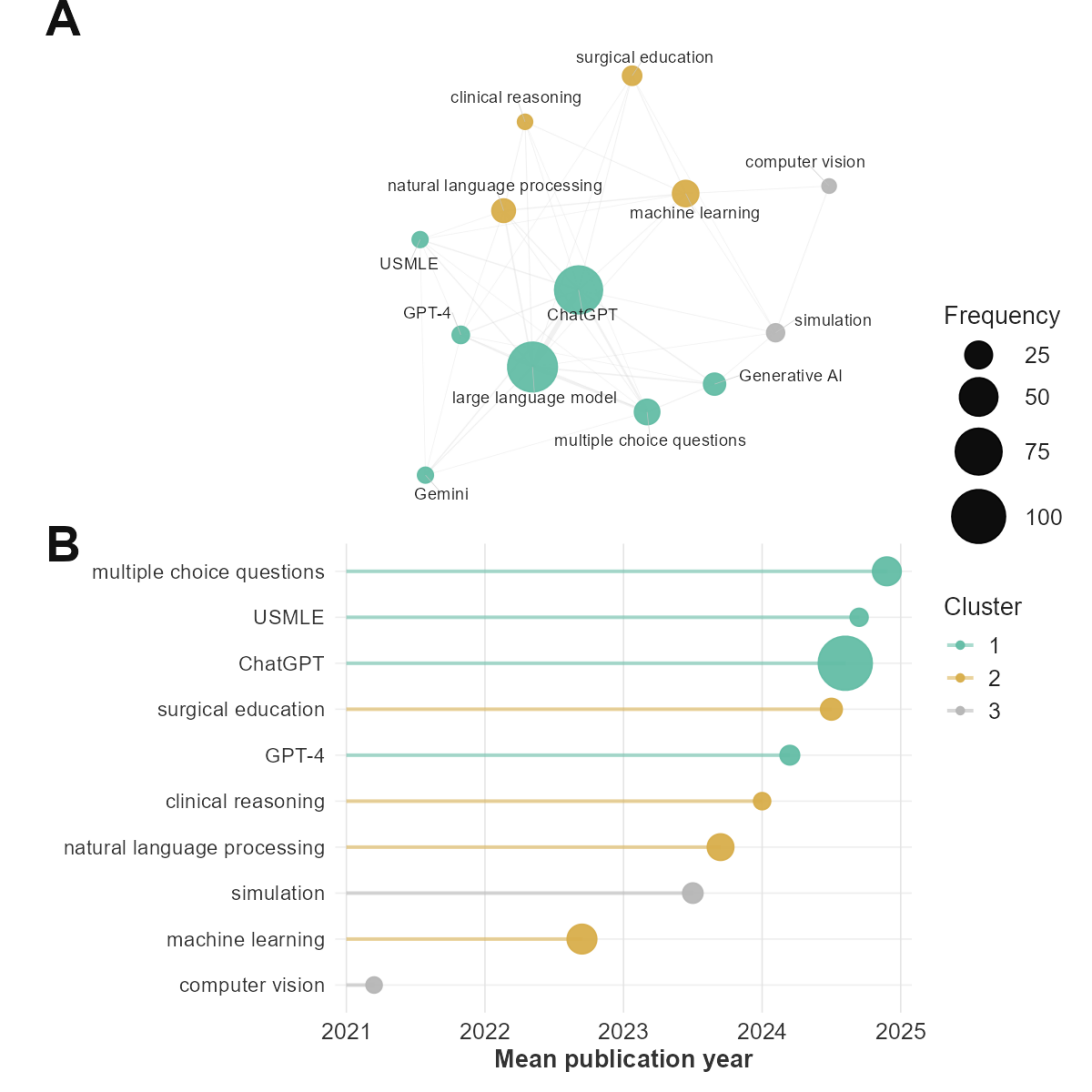
Keyword structure and thematic evolution of the literature. Panel A shows the co-occurrence network of normalized higher-frequency keywords. Panel B shows temporal profiles of leading keywords across the study period, highlighting movement from earlier machine-learning and simulation themes toward recent large language model and generative artificial intelligence assessment themes.

The keyword structure reflected the field’s recent shift toward GenAI and examination-oriented assessment (Figure 5). Frequent normalized keywords included large language model (n=109, 25.1%), ChatGPT (n=101, 23.2%), machine learning (n=29, 6.7%), multiple-choice questions (n=27, 6.2%), and natural language processing (n=23, 5.3%). The co-occurrence structure showed a recent cluster centered on large language models, ChatGPT, GPT-4, multiple-choice questions, and the United States Medical Licensing Examination (USMLE), alongside clusters linking natural language processing or machine learning with clinical reasoning, surgical education, simulation, and computer vision. The recent post-ChatGPT co-citation heatmap showed a compact intellectual structure anchored by early examination-benchmarking papers and synthesis work, including studies by Kung et al [8], Gilson et al [9], and Sallam [7] (Multimedia Appendix 4). This pattern suggests that the recent assessment-focused literature has developed rapidly around a relatively concentrated set of GenAI and examination-benchmarking references.

### Evidence Map of Assessment Functions, Settings, and Learner Stages

Across the final manually reviewed evidence-map coding, GenAI was coded in 310 records (71.3%) and large language models in 301 records (69.2%). The most common assessment functions were summative assessment (n=168, 38.6%), feedback (n=93, 21.4%), question generation (n=59, 13.6%), and automated scoring (n=42, 9.7%).

To separate assessment content generation from learner-facing assessment activities, we added an assessment-function umbrella analysis. Learner performance evaluation accounted for 270 records (62.1%), feedback for 93 records (21.4%), assessment content generation for 65 records (14.9%), and other or unclear functions for 7 records (1.6%). Question generation and item generation were treated as assessment content generation and were not automatically interpreted as summative assessment unless explicitly linked to learner evaluation, examination use, or assessment decision-making.

The most common assessment settings were board-style examinations (n=151, 34.7%), written examinations (n=88, 20.2%), procedural skills (n=52, 12%), and simulation (n=41, 9.4%). Undergraduate medical education was the most represented learner stage (n=172, 39.5%), followed by residency (n=84, 19.3%) and mixed or multiple learner stages (n=73, 16.8%).

**Figure 6:**
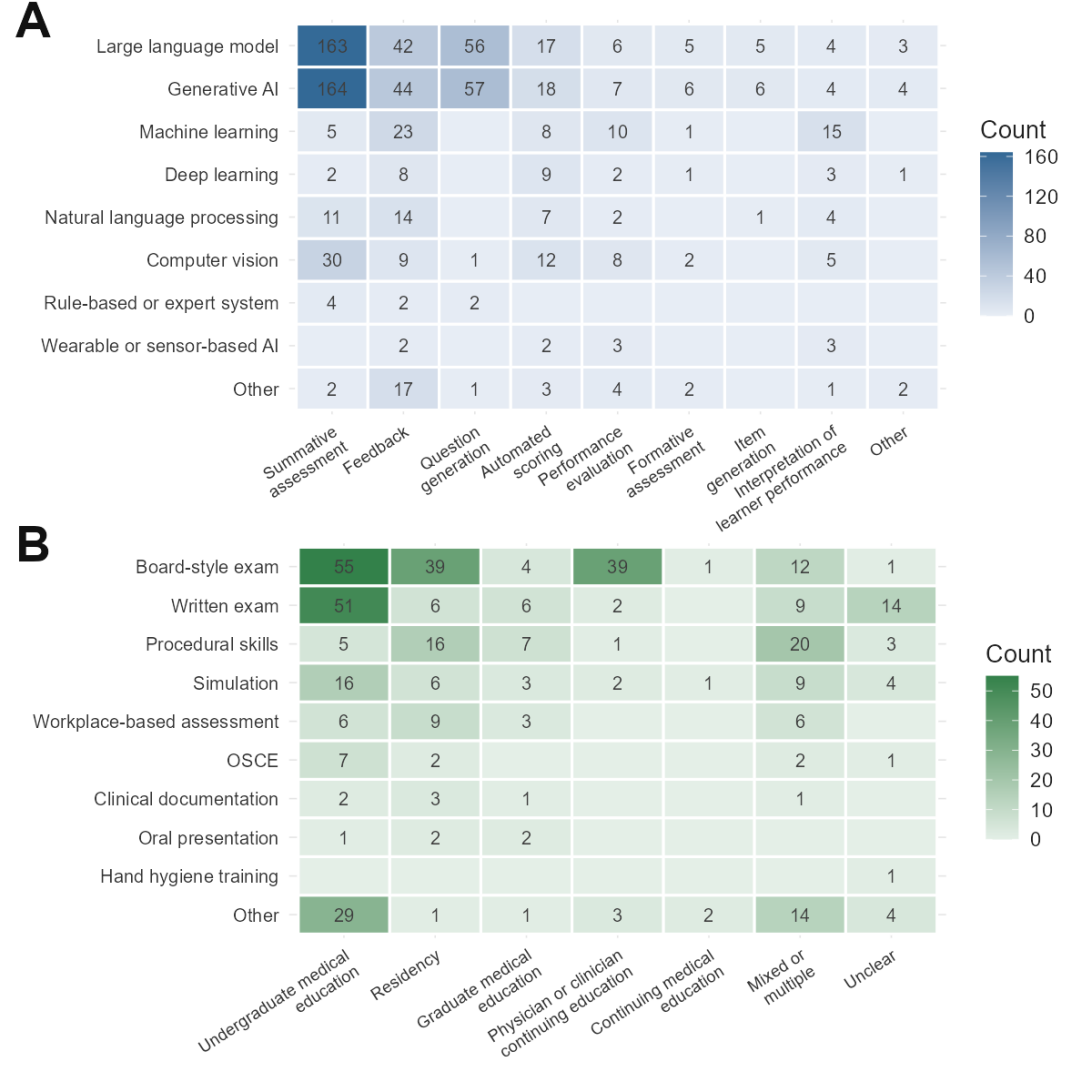
Evidence maps of assessment use. Panel A shows the assessment function by artificial intelligence type. Panel B shows the assessment setting by learner stage. Counts are based on nonmutually exclusive evidence-map coding categories and therefore represent coded study characteristics, not mutually exclusive study strata. Question generation and item generation were treated as assessment content generation and were not automatically interpreted as summative assessment unless explicitly linked to learner evaluation, examination use, or assessment decision-making.

The assessment function by AI type matrix showed concentration of GenAI and large language model records in summative assessment, question generation, and feedback (Figure 6). The largest intersections were GenAI with summative assessment (164 records), large language models with summative assessment (163 records), GenAI with question generation (57 records), large language models with question generation (56 records), and GenAI with feedback (44 records). The assessment setting by learner stage matrix showed that board-style and written examinations were concentrated most clearly in undergraduate medical education, with 55 records in the board-style examination by the undergraduate medical education cell and 51 records in the written examination by the undergraduate medical education cell. Board-style examination records also appeared in residency and physician or clinician continuing education, with 39 records in each of those cells.

### Full-Text Reporting-Domain Sensitivity Analysis

The reporting-domain variables visible in titles or abstracts were retained as the baseline. Because several reporting domains may be discussed mainly in full-text sections, we conducted a full-text sensitivity analysis across all 435 included records and used the full-text–confirmed estimates as the primary basis for interpreting reporting-domain findings.

**Figure 7:**
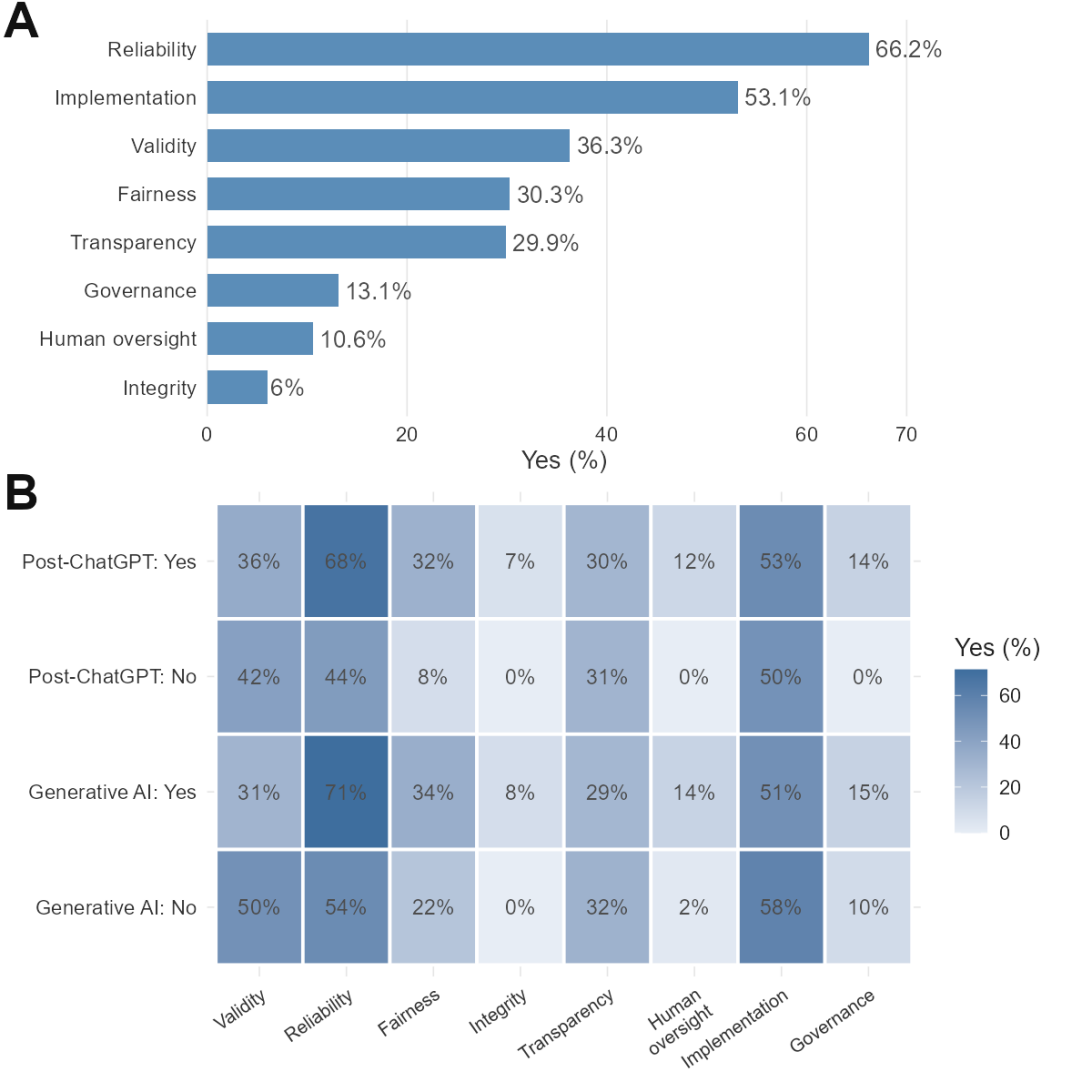
Full-text–confirmed reporting of assessment quality, implementation, and governance domains. Panel A shows the percentage of records with full-text–confirmed reporting for each domain. Panel B shows descriptive subgroup percentages by post-ChatGPT period and generative artificial intelligence relevance. Reporting-domain estimates are based on the full-text sensitivity analysis across all 435 included records. Subgroup percentages are descriptive; Fisher exact tests with multiplicity correction are reported in Multimedia Appendix 3.

In the full-text–confirmed analysis, reliability was identified in 288 (66.2%) records and implementation in 231 (53.1%) records (Figure 7). Validity, fairness, and transparency were identified at intermediate levels: validity in 158 (36.3%) records, fairness in 132 (30.3%) records, and transparency in 130 (29.9%) records. Governance, human oversight, and integrity were identified less frequently: governance in 57 (13.1%) records, human oversight in 46 (10.6%) records, and integrity in 26 (6%) records.

Compared with the baseline based on titles and abstracts, full-text–confirmed estimates increased for validity from 84 (19.3%) to 158 (36.3%), a net increase of 74 records; reliability from 107 (24.6%) to 288 (66.2%), a net increase of 181 records; fairness from 45 (10.3%) to 132 (30.3%), a net increase of 87 records; integrity from 11 (2.5%) to 26 (6%), a net increase of 15 records; transparency from 19 (4.4%) to 130 (29.9%), a net increase of 111 records; and implementation from 212 (48.7%) to 231 (53.1%), a net increase of 19 records. Human oversight remained unchanged at the aggregate level, with 46 records (10.6%) in both the baseline based on titles and abstracts and full-text–confirmed analysis. Governance changed from 62 records (14.3%) in the baseline based on titles and abstracts to 57 records (13.1%) in the full-text–confirmed analysis, a net decrease of 5 records after full-text adjudication.

These findings indicate that coding based on titles and abstracts underestimated several reporting domains, particularly reliability, transparency, fairness, and validity. The full-text sensitivity analysis refined the interpretation of reporting domains while leaving the main evidence-map categories based on the final manually reviewed coding based on titles and abstracts, with targeted ambiguity resolution.

### Coding Reliability

Stage 1 agreement for evidence-map categories coded from titles and abstracts was calculated across the full cohort of 435 records. Agreement was 393 (90.3%) for publication type, with Cohen κ of 0.785; 385 (88.5%) for study design, with Cohen κ of 0.843; 391 (89.9%) for AI type exact set, with Cohen κ of 0.859; 391 (89.9%) for assessment function exact set, with Cohen κ of 0.868; 392 (90.1%) for assessment-function umbrella, with Cohen κ of 0.826; 391 (89.9%) for assessment setting, with Cohen κ of 0.870; 400 (92%) for learner stage, with Cohen κ of 0.895; 407 (93.6%) for GenAI relevance, with Cohen κ of 0.851; and 423 (97.2%) for post-ChatGPT period, with Cohen κ of 0.834.

Stage 2 agreement for full-text reporting-domain coding was also calculated across the full cohort of 435 records. Agreement was 411 (94.5%) for validity, with Cohen κ of 0.877; 400 (92%) for reliability, with Cohen κ of 0.830; 414 (95.2%) for fairness, with Cohen κ of 0.880; 427 (98.2%) for integrity, with Cohen κ of 0.809; 413 (94.9%) for transparency, with Cohen κ of 0.873; 422 (97%) for human oversight, with Cohen κ of 0.819; 404 (92.9%) for implementation, with Cohen κ of 0.858; and 420 (96.6%) for governance, with Cohen κ of 0.830. For low-prevalence domains such as integrity and human oversight, percent agreement was interpreted alongside Cohen κ because prevalence can influence κ estimates.

### Subgroup and Partial-Year Sensitivity Analyses

Exploratory subgroup analyses for all 8 full-text–confirmed reporting domains are reported in Multimedia Appendix 3. Fisher exact tests with Benjamini-Hochberg correction were used to support these comparisons. In the main text, subgroup findings are summarized selectively and should be interpreted cautiously as descriptive and noncausal.

In the GenAI subgroup comparison, GenAI-related records had higher full-text–confirmed reporting of reliability than records not coded as involving GenAI (221/310, 71.3% vs 67/125, 53.6%), fairness (105/310, 33.9% vs 27/125, 21.6%), integrity (26/310, 8.4% vs 0/125, 0%), and human oversight (43/310, 13.9% vs 3/125, 2.4%). Validity was identified less often in GenAI-related records than in records not coded as involving GenAI (95/310, 30.6% vs 63/125, 50.4%), and transparency was similar between groups (90/310, 29% vs 40/125, 32%). These subgroup differences may reflect differences in publication period, assessment context, and application type.

In the post-ChatGPT comparison, the pre-ChatGPT subset was small (36 records). Reliability, fairness, human oversight, and governance were identified more often in the post-ChatGPT subset, whereas transparency was similar across periods. Detailed counts, unadjusted *P* values, and multiplicity-adjusted *P* values are provided in Multimedia Appendix 3.

Because 2026 was a partial year through April 8, 2026, we conducted an excluding-2026 sensitivity analysis. Excluding the 65 records from 2026, there were 370 records. Full-text–confirmed reporting-domain estimates changed by 0 to 3.9 percentage points. The largest absolute change was for transparency, which changed from 130 of 435 (29.9%) in the full cohort to 96 of 370 (25.9%) after excluding 2026. The overall interpretation was unchanged: reliability and implementation were the most frequently identified domains, validity, fairness, and transparency were identified at intermediate levels, and integrity, human oversight, and governance remained less frequently identified.

## Discussion

### Principal Findings

This bibliometric mapping study and thematic evidence map showed rapid growth in the literature on AI for assessment and feedback in medical education, particularly after the public release of ChatGPT. The mapped literature was concentrated in GenAI and large language model applications, with prominent representation of summative assessment, board-style examinations, written examinations, and undergraduate medical education.

The full-text sensitivity analysis showed uneven reporting of assessment-quality, implementation, and governance domains: reliability and implementation were frequently identified; validity, fairness, and transparency were identified at intermediate levels; and integrity, human oversight, and governance remained less frequently identified. The full-text sensitivity analysis also showed that coding based on titles and abstracts underestimated several reporting domains, supporting the decision to interpret reporting-domain findings primarily using full-text–confirmed estimates while retaining estimates based on titles and abstracts as the baseline comparison.

### Assessment Focus, Learner Stages, and Educational Implications

The prominence of board-style examinations and written examinations is understandable in the context of GenAI. Standardized questions provide accessible test beds, defined answer structures, and clear scoring benchmarks. They also allow rapid comparison across models and versions. For this reason, examination-benchmarking and evaluation studies have been useful for demonstrating what large language models can and cannot do on constrained knowledge-assessment tasks [8-10,27,28]. These studies are valuable for defining technical questions, including model performance, response consistency, prompt sensitivity, and knowledge retrieval.

However, benchmark performance alone is not sufficient to guide AI use in medical education assessment. Authentic assessment often requires observing learners in context, interpreting performance over time, integrating multiple sources of evidence, and making judgments that are educationally and professionally consequential [11-15]. Workplace-based assessment, procedural skills assessment, simulation, entrustment-related judgment, clinical reasoning assessment, and longitudinal feedback depend on context, supervision, relationships, and human interpretation in ways that differ from multiple-choice examination performance [29-33]. In the present map, board-style and written examinations were more common than procedural skills and simulation. This distribution suggests that the current assessment-focused AI literature is more developed for structured examination contexts than for assessment environments that require contextual judgment and integration of human observation.

The learner-stage distribution further reinforces this interpretation. Undergraduate medical education was the most represented learner stage, followed by residency and mixed or multiple learner stages. Undergraduate contexts are important sites for AI literacy, early clinical reasoning, and examination preparation [6,34-37]. At the same time, AI-supported assessment in residency, graduate medical education, continuing medical education, and clinician continuing professional development may raise different questions about workplace performance, entrustment, certification, supervision, and accountability. Future work should therefore examine AI-supported assessment across the continuum of medical training without assuming that evidence from undergraduate examination contexts generalizes to all learner stages.

The distinction between assessment content generation and learner-facing assessment is also central. AI-generated questions, cases, rubrics, or feedback templates may support educators and assessment systems, but producing assessment content is not the same as evaluating learner performance. Learner-facing assessment involves judging what a learner knows or can do, determining what feedback is appropriate, and sometimes informing progression or remediation. Therefore, the assessment-function disaggregation helps clarify the structure of the field. Assessment content generation may require evidence of item quality, alignment, difficulty, bias, and review processes [11-16,38]. Learner-facing scoring or feedback requires evidence about validity, reliability, fairness, transparency, human oversight, and educational consequences [16-20].

Feedback is a particularly important area for future development. AI-supported feedback could provide timely explanations, identify patterns in learner performance, draft individualized comments, or help educators manage feedback workload. Yet feedback is not only information delivery; it depends on learner interpretation, credibility, relationship, timing, and opportunities for improvement [39-41]. For learner-facing AI assessment or feedback studies that function as educational interventions, educational-intervention reporting guidance such as GREET (Guideline for Reporting Evidence-Based Practice Educational Interventions and Teaching) may help clarify the educational context, intervention components, implementation conditions, learner outcomes, and feedback processes [42]. The most educationally defensible uses of AI feedback may therefore be hybrid workflows in which AI supports generation, organization, or pattern recognition while educators retain responsibility for contextualization, escalation, and high-stakes judgment. This framing positions AI as a tool that may augment feedback processes when appropriately validated and governed [43].

### Full-Text Reporting Domains, Governance, and Responsible Assessment

The full-text sensitivity analysis refined the reporting-domain findings. In the baseline based on titles and abstracts, several domains appeared uncommon. After full-text review across all 435 records, reliability and implementation were frequently identified; validity, fairness, and transparency were identified at intermediate levels; and integrity, human oversight, and governance remained less frequent. This pattern supports a more nuanced interpretation: reporting was uneven, not uniformly sparse.

This distinction matters because assessment places particular demands on responsible AI use. Reliability and implementation are necessary, but they are not sufficient. Validity is needed to support the intended interpretation and use of assessment results [11-16,38]. Fairness, transparency, and human oversight are also needed so educators and learners can understand, question, and appropriately rely on AI-supported outputs [17-20]. The intermediate full-text reporting of validity, fairness, and transparency suggests that these issues are present in the literature, but they are not yet consistently integrated across assessment-focused studies.

The lower reporting of integrity, human oversight, and governance is particularly relevant for medical education assessment. Integrity concerns whether AI changes what is being assessed, creates opportunities for misuse, or undermines confidence in the interpretation of learner performance. Human oversight concerns who reviews AI outputs, when educators intervene, and how disagreements or uncertain outputs are handled. Governance concerns institutional policy, accountability, privacy, data governance, acceptable use, escalation, and monitoring [17-20,43]. These domains are especially important when AI-supported systems contribute to feedback, scoring, performance interpretation, or progression-related judgments.

The subgroup analyses were exploratory and should not be interpreted causally. GenAI-related records differed from records not coded as involving GenAI in the publication period, assessment context, and application type, and the pre-ChatGPT subset was small. Still, the descriptive patterns reinforce the need for more explicit reporting as the field matures. In assessment contexts, responsible AI reporting is not an administrative add-on. It shapes whether a tool can be trusted, how it should be supervised, what decisions it can support, and how learners and educators can challenge or interpret its outputs.

### Comparison With Prior Work

This study builds on broad reviews and bibliometric analyses of AI in medical education, which have described rapid growth in AI-related educational scholarship and highlighted applications in curriculum design, simulation, clinical reasoning, feedback, learner support, and institutional readiness [3,6,21,22]. Those studies are essential for understanding the wider educational landscape. The present study contributes a narrower but complementary view by isolating assessment and feedback.

This assessment-specific focus changes what becomes visible. In broad AI-in-medical-education syntheses, assessment may appear as one application among many. In the present map, assessment-related work shows a distinct profile: strong post-ChatGPT growth, concentration in GenAI and large language models, prominence of examination-oriented settings, and comparatively less representation of authentic assessment contexts such as workplace-based assessment, procedural assessment, and longitudinal performance interpretation. The evidence-map approach also highlights the importance of separating assessment content generation from learner-facing evaluation and feedback.

The full-text reporting-domain analysis also extends prior work. Responsible AI and ethical guidance in health professions education have emphasized fairness, transparency, accountability, human involvement, privacy, and governance [17-20,43]. Our results show how often related domains are explicitly identifiable in the assessment-focused literature. The finding that reliability and implementation were frequently identified, validity, fairness, and transparency were intermediate, and integrity, human oversight, and governance were less frequent, provides a more granular view than broad claims about underreporting across all domains.

Finally, the bibliometric component complements prior field-level studies by showing how the assessment-focused subfield is structured around recent GenAI and examination-benchmarking work. This is useful for medical educators and researchers because it identifies both the current center of activity and areas where the evidence base remains less developed. Future assessment-focused studies can build on benchmark work while expanding into authentic performance contexts, learner development, faculty judgment, and governance of AI-supported assessment systems.

### Limitations

This study has several limitations. First, the search was limited to Web of Science Core Collection, Scopus, and PubMed, and to English-language records. Relevant publications indexed only in other databases or published in other languages may have been missed. Country and institution findings should therefore be interpreted as patterns within the indexed English-language literature captured by the search, not as complete global research output.

Second, this was a bibliometric mapping study with structured evidence-map coding. The study maps the literature and its reported characteristics but does not evaluate intervention effectiveness, compare educational outcomes, or assess risk of bias for individual empirical studies.

Third, document selection and the main evidence-map categories were based primarily on titles, abstracts, and bibliographic metadata, with targeted ambiguity resolution when needed. This approach was aligned with the bibliographic-record-based mapping purpose of the study, but some records may have been incompletely characterized at the level of titles and abstracts. To address this limitation for domains especially likely to appear outside abstracts, we added a full-text sensitivity analysis for validity, reliability, fairness, integrity, transparency, human oversight, implementation, and governance across all 435 included records.

Fourth, evidence-map categories were nonmutually exclusive. This was appropriate for representing multidimensional records that involved more than 1 AI type, assessment function, setting, or learner stage, but counts should not be interpreted as mutually exclusive study strata. Fifth, subgroup analyses were exploratory. Although Fisher exact tests with Benjamini-Hochberg correction were conducted, subgroup patterns may reflect differences in publication period, study type, assessment context, or AI application and should not be interpreted as independent associations.

Sixth, citation-based indicators are influenced by publication timing, journal indexing, database coverage, and the short time window since the release of ChatGPT. Recent records may not yet have accumulated citations, whereas early benchmark papers may appear especially prominent because they became early anchors for subsequent work. Finally, 2026 was a partial year through April 8, 2026. We marked 2026 as partial in the annual trend figure and conducted an excluding-2026 sensitivity analysis; the main interpretation of reporting-domain patterns was unchanged.

### Conclusions

The literature on AI for assessment and feedback in medical education has expanded rapidly, particularly in the post-ChatGPT period. Within the mapped indexed English-language literature, research was concentrated in GenAI, large language models, summative assessment, board-style and written examinations, and undergraduate medical education. The full-text sensitivity analysis showed that reporting of assessment-quality, implementation, and governance domains was uneven: reliability and implementation were frequently identified; validity, fairness, and transparency were identified at intermediate levels; and integrity, human oversight, and governance remained less frequent.

Future research should complement examination benchmarking with more authentic assessment contexts, including workplace-based assessment, simulation, procedural skills assessment, longitudinal feedback, and performance interpretation across the continuum of medical training. Studies should distinguish assessment content generation from learner-facing evaluation and feedback, and should report more consistently on validity, fairness, transparency, integrity, human oversight, implementation, and governance. For medical education assessment, the central question is not only whether AI can perform isolated assessment tasks, but also how AI-supported workflows can improve learning and assessment while preserving accountability, fairness, and human educational judgment.

## Data Availability

Summary data supporting the findings are provided in the article and the Multimedia Appendices. Because the source records were obtained from third-party bibliographic databases subject to their respective licensing terms, raw database exports are not publicly shared. Additional derived summary tables are available from the corresponding author on reasonable request, subject to database licensing restrictions. The R scripts used to generate the derived summary tables, sensitivity analyses, agreement analyses, subgroup analyses, partial-year sensitivity analyses, and figures will be made publicly available in a GitHub repository after acceptance.

## Appendix

Multimedia Appendix 1 Completed BIBLIO reporting checklist. Completed checklist showing where BIBLIO reporting items are addressed in the revised manuscript and supplementary materials.

Multimedia Appendix 2 Detailed search strategies for Web of Science Core Collection, Scopus, and PubMed. Database-specific search strings; search limits; final search date of April 8, 2026; search period from January 1, 2015, to April 8, 2026; and final hit counts.

Multimedia Appendix 3 Supplementary methods and results. Supplementary methodological reporting, coding definitions, bibliometric software and parameter settings, full-text sensitivity coding rules, study selection and annual trend details, descriptive evidence-map tables, assessment-function disaggregation, full-text reporting-domain summaries, reporting shifts from estimates visible in titles or abstracts to full-text-confirmed estimates, two-stage agreement analyses, subgroup Fisher exact tests with Benjamini-Hochberg correction, excluding-2026 partial-year sensitivity analysis, and bibliometric summaries.

Multimedia Appendix 4 Supplementary co-citation heatmap of the recent post-ChatGPT assessment literature. Co-citation heatmap showing the recent intellectual structure of the post-ChatGPT literature on artificial intelligence for assessment and feedback in medical education.

## Abbreviations

AI: artificial intelligence
BIBLIO: bibliometric reviews of biomedical literature
GenAI: generative artificial intelligence
GREET: Guideline for Reporting Evidence-Based Practice Educational Interventions and Teaching
USMLE: United States Medical Licensing Examination
