# Supplementary material for: Artificial Intelligence for Assessment and Feedback in Medical Education: Bibliometric Mapping Study and Thematic Evidence Map
Source: JMIR Med Educ. 2026 Jul 2;12:e98949. doi: 10.2196/98949 (PMC13376851; doi:10.2196/98949)
Supplement: Multimedia Appendix 1 [file mededu_v12i1e98949_app1.docx]

Multimedia Appendix 1. Completed BIBLIO Reporting Checklist

*Artificial Intelligence for Assessment and Feedback in Medical Education: A Bibliometric Mapping Study and Thematic Evidence Map*

Page numbers refer to the clean revised manuscript prepared for resubmission. The checklist is completed using the BIBLIO checklist where applicable to a bibliometric mapping study incorporating structured thematic evidence-map coding.

| **Section** | **Topic** | **Item No.** | **Checklist item** | **Reported on page No. / location** |
| --- | --- | --- | --- | --- |
| Title | Identification | 1 | Identify the report as a bibliometric review in the title. | Page 1: title identifies the study as a “Bibliometric Mapping Study and Thematic Evidence Map.” |
| Title | Issues/topics | 2 | Indicate the key issues/topics under investigation and coverage of time period. | Pages 1-2: title and structured abstract state AI for assessment and feedback in medical education; page 4: Methods specify the search period from January 1, 2015, to April 8, 2026. |
| Abstract | Structured summary | 3 | Structured summary including (as applicable): background, methods, results (key findings) and conclusions. | Pages 1-2: Structured Abstract includes Background, Objective, Methods, Results, and Conclusions. |
| Introduction/Background | Justification / Rationale / Explanation | 4 | Present review of existing knowledge and epidemiological information. | Pages 2-4: Introduction summarizes existing AI in medical education literature, generative AI and large language models, assessment and feedback rationale, and gaps in assessment-specific evidence. |
| Introduction/Background | Objectives | 5 | Statement of the objective(s) or question(s). | Pages 3-4: final Introduction paragraph states the study aim and specific mapping objectives. |
| Methods | Search engines (data sources) | 6 | Describe all information sources (such as electronic databases, contact with study authors, trial registers or other grey literature sources). | Page 4: Data Sources and Search Strategy reports Web of Science Core Collection, Scopus, and PubMed; Multimedia Appendix 2 provides database-specific search strategies. |
| Methods | Search strategy | 7 | Keywords and systematization criteria (date of search, language, type of document) for the search. | Pages 4-5: search concepts, final search date, and English-language/indexing criteria are reported; Multimedia Appendix 2 provides complete database-specific search strings, limits, and final hit counts. |
| Methods | Time period | 8 | The period that the review covers and the justification. | Pages 4 and 8: Methods report January 1, 2015, to April 8, 2026, justify the three-database strategy, and define the post-ChatGPT period as November 2022 onward. |
| Methods | Eligibility criteria | 9 | Describe all inclusion and exclusion criteria; languages; study design, type of publication and time period. | Pages 5-6: Eligibility Criteria describes included assessment/feedback contexts, educational populations, publication types, exclusions, English-language restriction, and selected databases. |
| Methods | Data refinement (data selection procedure) | 10 | Remove the irrelevant articles; inspection to eliminate duplicate and unrelated articles (after evaluation of the title, abstract and content). | Pages 5-6 and 9: Deduplication and Document Selection describe multistage deduplication, title/abstract-level selection, targeted ambiguity resolution, and final cohort; page 9: Results provide search and selection counts and Figure 1 provides the flow diagram. |
| Methods | Quality assessment (optional) | 11 | Assessment of papers by three authors and the use of assessing checklists. | Pages 6-8 and 15-17: not applicable as a risk-of-bias or intervention-effectiveness assessment; coding reliability, full-text sensitivity analysis, and adjudication procedures are reported, with detailed agreement tables in Multimedia Appendix 3. |
| Methods | Data synthesis | 12 | Describe the methods used for summarizing, handling, synthesis, tabulations or schematic displays. Describe how the data were analysed. | Pages 6-9: evidence-map coding, full-text sensitivity analysis, coding reliability, bibliometric analysis, evidence-map matrices, subgroup analyses, and partial-year sensitivity analyses are described; Multimedia Appendix 3 reports coding definitions, bibliometric parameters, subgroup analyses, and supplementary tables. |
| Results | Descriptive findings (statistics) | 13 | Provide details of the search and selection process in a flow diagram; report number of citations retrieved and descriptive characteristics such as publication year, type of documents, countries, authors, journals, institutions, and other relevant indicators. | Pages 9-17: study selection, temporal growth, journal landscape, country collaboration, institutions, authors, keywords, evidence-map findings, reporting domains, coding reliability, subgroup analyses, and partial-year sensitivity are reported; Figure 1 provides the selection flow diagram. |
| Results | Schematic map and trend | 14 | Summarize and/or present the schematic maps and trends using appropriate software to present citations, journals, authors, top journals, time trends, emerging literature, and relevant indicators. | Pages 9-17: Results summarize annual trends, journal landscape, collaboration/contributor structure, keyword structure, evidence maps, reporting-domain patterns, coding reliability, and sensitivity analyses; Figures 1-7 and Multimedia Appendices 3-4 provide visual and supplementary outputs. |
| Results | Tabulation and summarizing the findings | 15 | Summarize and organize the findings by relevant subtitles, scenarios, study designs, populations, concepts, or topics as appropriate. | Pages 9-17: Results are organized by study selection and temporal growth, bibliometric structure and publication characteristics, assessment functions/settings/learner stages, full-text reporting-domain sensitivity analysis, coding reliability, and subgroup/partial-year sensitivity analyses; Multimedia Appendix 3 provides supplementary tables. |
| Results | Synthesis of findings | 16 | Synthesize the findings as much as possible, find the gap, and propose a model, hypothesis, etc. (if applicable). | Pages 17-22: Discussion synthesizes findings around examination benchmarking, authentic assessment, learner stages, assessment content generation versus learner-facing assessment, feedback, responsible AI reporting domains, governance, and comparison with prior work. |
| Discussion | Summary of evidence | 17 | Summarize the main findings. The findings should be presented in more general or accessible terms. | Pages 17-18: Principal Findings summarize rapid growth, concentration in generative AI/large language models and examination-oriented settings, and uneven full-text-confirmed reporting domains. |
| Discussion | Interpretation | 18 | Include interpretation consistent with results. Explanations for observed outcomes, similarities, and differences reported would be essential. | Pages 18-22: Discussion interprets examination benchmarking, authentic assessment, learner-stage distribution, assessment-function disaggregation, feedback, full-text reporting domains, subgroup patterns, and comparison with prior work. |
| Discussion | Strengths and limitations | 19 | Discuss the strengths and limitations. | Pages 4-9 and 15-17: methodological strengths are addressed through full-text sensitivity analysis, two-stage coding reliability, subgroup analyses, partial-year sensitivity analysis, and supplementary bibliometric parameters; pages 21-22: Limitations discuss database/language coverage, mapping design, title/abstract-level coding, nonmutually exclusive categories, exploratory subgroup analyses, citation timing, and partial-year 2026. |
| Discussion | Conclusion(s) | 20 | Provide a general interpretation of the results with respect to the review questions and objectives, as well as potential implications. | Page 22: Conclusions summarize findings and implications for future assessment-focused AI research, including authentic assessment contexts, assessment-function distinction, and responsible reporting domains. |

Rights and permissions: The checklist was adapted from Montazeri A, Mohammadi S, M.Hesari P, Ghaemi M, Riazi H, Sheikhi-Mobarakeh Z. Preliminary guideline for reporting bibliometric reviews of the biomedical literature (BIBLIO): a minimum requirements. Systematic Reviews. 2023;12:239. doi:10.1186/s13643-023-02410-2. The original article is licensed under a Creative Commons Attribution 4.0 International License (http://creativecommons.org/licenses/by/4.0/).
