# Supplementary material for: Artificial Intelligence for Assessment and Feedback in Medical Education: Bibliometric Mapping Study and Thematic Evidence Map
Source: JMIR Med Educ. 2026 Jul 2;12:e98949. doi: 10.2196/98949 (PMC13376851; doi:10.2196/98949)
Supplement: Multimedia Appendix 2 [file mededu_v12i1e98949_app2.docx]

**Multimedia Appendix 2. Detailed search strategies for Web of Science Core Collection, Scopus, and PubMed**

**Search date:** 2026-04-08

**Web of Science Core Collection**

**Search entry:** Advanced Search

**Search string:**
TS=(("artificial intelligence" OR "machine learning" OR "deep learning" OR "generative artificial intelligence" OR "generative AI" OR "large language model*" OR LLM* OR ChatGPT OR GPT OR "foundation model*" OR "language model*") AND (assessment OR evaluation OR feedback OR scoring OR grading OR examination* OR exam* OR test* OR testing OR OSCE OR "objective structured clinical examination" OR rubric* OR "item generation" OR "question generation" OR "performance evaluation" OR "automated scoring" OR "formative assessment" OR "summative assessment") AND ("medical education" OR "medical student*" OR "undergraduate medical education" OR "graduate medical education" OR resident* OR residency OR clerkship* OR internship OR "continuing medical education" OR "continuing professional development" OR CME OR CPD))

**Limits applied:** Publication years from 2015-01-01 to 2026-04-08; document types limited to article or review; language limited to English.

**Final hit count after filters:** 4381

**Scopus**

**Search entry:** Advanced Search

**Search string:**
TITLE-ABS-KEY((("artificial intelligence" OR "machine learning" OR "deep learning" OR "generative artificial intelligence" OR "generative AI" OR "large language model*" OR LLM* OR ChatGPT OR GPT OR "foundation model*" OR "language model*") AND (assessment OR evaluation OR feedback OR scoring OR grading OR examination* OR exam* OR test* OR testing OR OSCE OR "objective structured clinical examination" OR rubric* OR "item generation" OR "question generation" OR "performance evaluation" OR "automated scoring" OR "formative assessment" OR "summative assessment") AND ("medical education" OR "medical student*" OR "undergraduate medical education" OR "graduate medical education" OR resident* OR residency OR clerkship* OR internship OR "continuing medical education" OR "continuing professional development" OR CME OR CPD)))

**Limits applied:** Publication years from 2015-01-01 to 2026-04-08; document types limited to article or review; language limited to English.

**Final hit count after filters:** 7256

**PubMed**

**Search entry:** Advanced Search

**Search string:**
(("artificial intelligence"[tiab] OR "machine learning"[tiab] OR "deep learning"[tiab] OR "generative artificial intelligence"[tiab] OR "generative AI"[tiab] OR "large language model*"[tiab] OR LLM[tiab] OR LLMs[tiab] OR ChatGPT[tiab] OR GPT[tiab] OR "foundation model*"[tiab] OR "language model*"[tiab]) AND (assessment[tiab] OR evaluation[tiab] OR feedback[tiab] OR scoring[tiab] OR grading[tiab] OR examination*[tiab] OR exam*[tiab] OR test*[tiab] OR testing[tiab] OR OSCE[tiab] OR "objective structured clinical examination"[tiab] OR rubric*[tiab] OR "item generation"[tiab] OR "question generation"[tiab] OR "performance evaluation"[tiab] OR "automated scoring"[tiab] OR "formative assessment"[tiab] OR "summative assessment"[tiab]) AND ("medical education"[tiab] OR "medical student*"[tiab] OR "undergraduate medical education"[tiab] OR "graduate medical education"[tiab] OR resident*[tiab] OR residency[tiab] OR clerkship*[tiab] OR internship[tiab] OR "continuing medical education"[tiab] OR "continuing professional development"[tiab] OR CME[tiab] OR CPD[tiab])) AND ("2015/01/01"[Date - Publication] : "2026/04/08"[Date - Publication]) AND english[lang] AND (journal article[pt] OR review[pt])

**Limits applied:** Publication date from 2015-01-01 to 2026-04-08; language limited to English; publication type limited to journal article or review.

**Final hit count after filters:** 3331

**Note:** In annual trend reporting, 2026 was treated as a partial year up to the search date.
