# Supplementary figures and images for: Artificial Intelligence for Assessment and Feedback in Medical Education: Bibliometric Mapping Study and Thematic Evidence Map
Source: JMIR Med Educ. 2026 Jul 2;12:e98949. doi: 10.2196/98949 (PMC13376851; doi:10.2196/98949)

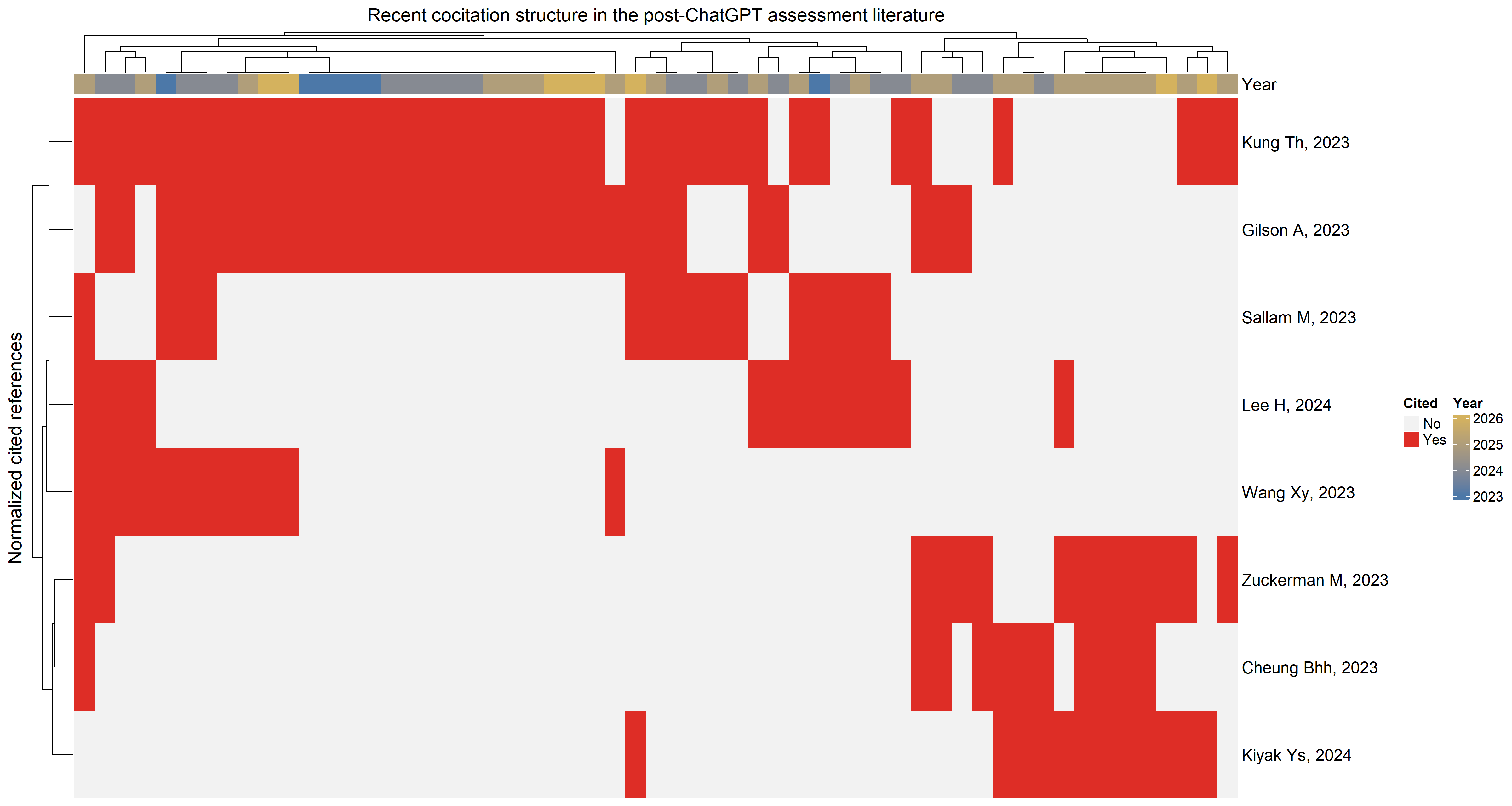

Supplement: Multimedia Appendix 4 [file mededu_v12i1e98949_app4.png]
